# The LNK adaptor protein: a dual regulator of proliferation and migration in solid tumors

**DOI:** 10.1080/23723556.2026.2644701

**Published:** 2026-03-16

**Authors:** Jessica C. Mantilla-Ollarves, Laura Velazquez, Leticia Rocha-Zavaleta

**Affiliations:** a Departamento de Biología Molecular y Biotecnología, Instituto de Investigaciones Biomédicas, Universidad Nacional Autónoma de México, Ciudad de México, México; b Programa de Doctorado en Ciencias Bioquímicas, Universidad Nacional Autónoma de México, Ciudad de México, México; c INSERM UMR-1342, Institut de Recherche Saint-Louis, Hôpital Saint-Louis, Paris, France

**Keywords:** LNK, JAK/STAT, proliferation, migration, solid tumors

## Abstract

Cancer is a significant public health problem. Alterations in critical cellular responses, such as proliferation, survival, and migration, are hallmarks of cancer. Deregulation of the JAK/STAT, MAPK, and PI3K/AKT signaling pathways has been implicated in the development of most solid cancers; therefore, their strict control is a primary research focus. The LNK adaptor protein is a key negative regulator of these pathways in normal cells and in certain hematological malignancies, where it regulates the proliferation, migration, and inflammatory response of different cell types. However, the function of LNK in solid tumors is less clear. Recent evidence suggests that LNK inhibits proliferation and migration in some types of solid tumors but activates these same processes in others. In this review, we provide an overview of the essential functions of LNK in healthy tissues, compare these functions with LNK’s reported activities in solid tumors, and discuss the potential mechanisms involved in the dual actions of the LNK protein.

## Introduction

Cancer is a major public health problem. According to GLOBOCAN estimates, over 20 million new cases were diagnosed in 2022, and this number may increase to nearly 30 million cases by 2040.^1^ For this reason, the management of cancer is expected to remain a social challenge and a significant economic threat, especially in less developed countries.^1^ Cancers can be divided into hematological malignancies and solid tumors, with the latter representing about 90% of cases.^2^ According to the age-standardized incidence rate reported by the World Health Organization, breast, prostate, lung, colorectal, and cervical cancer are the top five cancer types globally.^1^

Although cancer is a multifactorial disease, and several risk factors are associated with the development of particular types of solid neoplasias, most tumors share molecular alterations in key signaling pathways controlling cell proliferation, migration, and inflammation. Among them, mitogen-activated protein kinase (MAPK), phosphatidylinositol 3-kinase (PI3K)/protein kinase B (AKT), and Janus kinase (JAK)/signal transducer and activator of transcription (STAT) are the most frequently altered signaling cascades. MAPKs regulate cell proliferation, differentiation, and survival,^3^ are associated with migration and invasion in colorectal cancer,^4^ and have been linked to proliferation and migration in breast cancer.^5^^,^^6^ Moreover, approximately 40% of human cancers have mutations or alterations in one or more MAPK-encoding genes, leading to hyperactivation of the signaling cascade.^7^ Additionally, the PI3K pathway plays a crucial role in cell survival and migration, as well as angiogenesis, in normal tissues, and it is commonly deregulated in breast cancer, colorectal cancer, glioblastoma, and endometrial cancer.^8^ Finally, the JAK/STAT pathway plays a crucial role in highly regulated processes such as hematopoiesis, mammary gland development, and immune activation.^9^ Thus, constitutive activation of this pathway promotes cancer cell proliferation, survival, and drug resistance.^10^ Because of the central nature of their activities, these signaling pathways are tightly regulated by specific molecules, such as the adapter protein LNK.

LNK, also known as Src homology-2 B3 (SH2B3), was first described as an essential controller of normal hematopoiesis, regulating proliferation of hematopoietic stem and progenitor cells, as well as of lymphoid and myeloid lineages in the bone marrow.^11^ LNK is also a negative regulator in non-hematopoietic cells, including endothelial cells (ECs),^12^ mesenchymal stem cells,^13^ neural cells,^14^ and osteoblasts.^15^ LNK can inhibit JAK tyrosine kinase activity,^16^ thereby preventing activation of downstream STAT transcription factors.^16^ It can also regulate AKT and ERK activation.^11^^,^^17^ Likewise, LNK mediates blockage of aberrant signaling caused by hyperactivation of molecules such as AKT, ERK, JAK2, and STAT3 in myeloproliferative neoplasms and leukemias.^18^^-^^21^

Notwithstanding, the role of LNK in solid tumors remains controversial. Some reports suggest that LNK overexpression promotes proliferation in breast cancer,^22^ glioblastoma,^23^ melanoma,^24^ ovarian cancer,^25^ and thyroid cancer,^26^ while others found that LNK inhibits proliferation and migration of lung cancer cells^27^ and invasion of colorectal cancer cells.^28^ Therefore, although LNK is a negative regulator of signaling pathways that are frequently deregulated in solid neoplasias, the potential contribution of LNK to the development of solid tumors is still unclear.

The aim of this review is to delve deeper into the functions of LNK in solid tumors, comparing them with those in normal tissue, and to propose mechanisms to explain the dual role this adapter molecule may play in controlling proliferation and migration in solid tumors.

## LNK inhibits multiple biological processes under physiological conditions

The LNK protein is encoded by the *SH2B3* gene, which was first cloned from a cDNA library constructed from rat lymph nodes.^29^ The human *LNK* gene was identified and characterized 5 y later, and it was found to share similarities with two other adapter proteins, SH2-B (SH2B1) and APS (SH2B2).^30^ For that reason, the three proteins were included within the SH2B adapter protein family, and LNK was named SH2B3.

### Role of LNK in regulating the JAK/STAT, MAPK, and PI3K/AKT pathways

**Figure 1.**
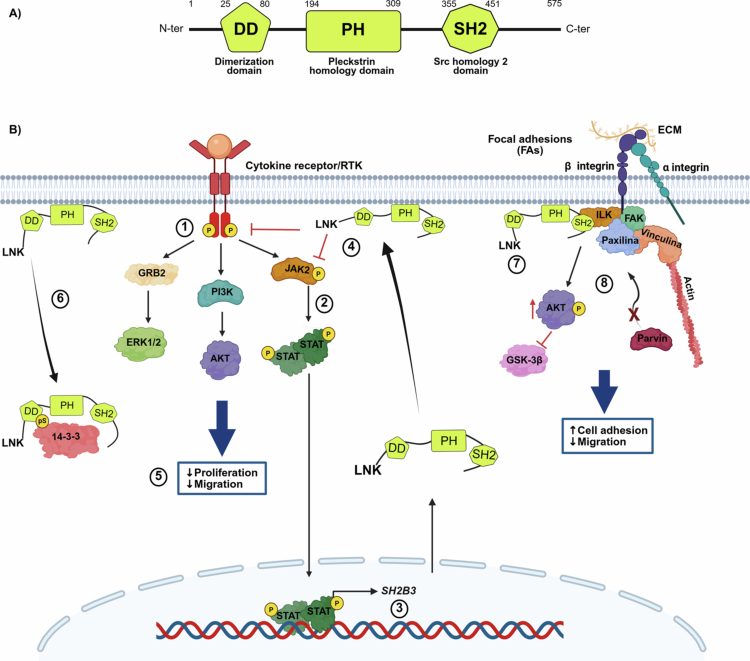
LNK structure and role in regulating the JAK/STAT, MAPK, PI3K/AKT, and integrin pathways in normal cells. (A) Main LNK domains. *N*-ter: amino terminus; DD: dimerization domain; PH: pleckstrin homology domain; SH2: Src homology 2 domain; C-ter: carboxy terminus. (B) LNK role in signaling pathways. (1) After binding to specific ligands, cytokine receptors and receptor tyrosine kinases activate the JAK/STAT, MAPK, and PI3K/AKT pathways. (2) JAK2 kinase phosphorylates and activates STAT proteins. (3) STAT dimers translocate to the cell nucleus and promote transcription of several genes, including negative pathway regulators such as SH2B3, which encodes LNK. (4) Once synthesized, LNK translocates to the inner face of the cytoplasmic membrane, where it binds to phosphorylated JAK2 and inhibits its function, thus creating a negative regulation loop. LNK can also regulate the MAPK and PI3K/AKT pathways. (5) Reduced proliferation and migration are associated with LNK activity. (6) LNK activity is negatively regulated by 14-3-3, which binds LNK at phosphorylated serine residues (pS) and prevents its translocation to the inner face of the cytoplasmic membrane. (7) When integrins recognize components of the extracellular matrix (ECM), LNK is recruited along with a protein complex to participate in the formation of focal adhesions (FAs). (8) The presence of LNK enables ILK activation and subsequent phosphorylation of AKT and GSK-3β. The ILK-LNK complex prevents ILK from interacting with *α*-parvin and inhibits FA disassembly. This improves adhesion and decreases migration. Created in https://BioRender.com.

LNK contains three functional domains: a dimerization domain at the *N*-terminus; a central pleckstrin homology (PH) domain that allows LNK to bind to the inner face of the cytoplasmic membrane by recognizing phosphatidylinositol lipids; and a Src homology 2 (SH2) domain at the C-terminus that recognizes phosphotyrosine residues in its target molecules (Figure 1A).^31^ In addition, phosphorylation of LNK at several tyrosine and serine residues further mediates its interactions with binding partners and effectors.^32^ Thus, LNK’s structure determines its intracellular localization, which is essential for carrying out its function within the cell. Some studies have shown that LNK is primarily located in the cytoplasm and at the cell membrane.^18^^,^^33^ Other reports have shown that LNK is present in the juxtanuclear region, but its function in this cellular compartment remains unclear.^12^^,^^30^

In normal cells, LNK activity is activated by cell signaling cascades that are stimulated by cytokines and growth factors. Most cytokine signaling pathways are triggered by activation of the receptor-associated JAK2 kinase.^34^ JAK2 phosphorylates STAT factors, which then translocate into the nucleus as dimers to induce the transcription of genes associated with proliferation and resistance to apoptosis.^34^ Interestingly, STAT1 induces *SH2B3* expression.^14^ The newly synthesized LNK then migrates from the cytoplasm to the inner face of the cell membrane, where it binds, through its SH2 domain, to activated JAK2^16^ or to activated receptor tyrosine kinases (RTKs) and blocks their tyrosine kinase activity, hindering downstream signaling.^35^^,^^36^ Thus, LNK is part of an inhibitory feedback loop that ensures precise downregulation of cytokine-activated pathways at specific times and in specific cellular spaces (Figure 1B).

Although LNK can inhibit both the MAPK and PI3K/AKT signaling cascades, the mechanism by which it exerts this effect is not completely understood. The MAPK and PI3K/AKT signaling pathways can be triggered by activated JAK2^34^ or by RTKs such as cKIT.^37^ Thus, it is possible that LNK indirectly regulates these signaling pathways by directly inhibiting RTKs and/or JAK2 activity.

While ample evidence supports the role of LNK as a negative regulator, the cellular mechanisms that modulate LNK activity are less well known. The only LNK regulator that has been reported in normal cells is 14-3-3.^33^ The 14-3-3 protein family comprises seven isoforms, all of which are expressed ubiquitously.^38^ Jiang et al. found that, in thrombopoietin-stimulated hematopoietic cells, 14-3-3 binds to and sequesters LNK in the cytoplasm, preventing it from trafficking to the cell membrane and inhibiting JAK2.^33^ They also demonstrated that LNK must be phosphorylated on residues S13 and S129 by GSK-3β and PKA, respectively, to bind 14-3-3.^33^ Other regulators still need to be identified to better understand how LNK is controlled in different cells.

### LNK functions in non-hematopoietic systems

**Figure 2.**
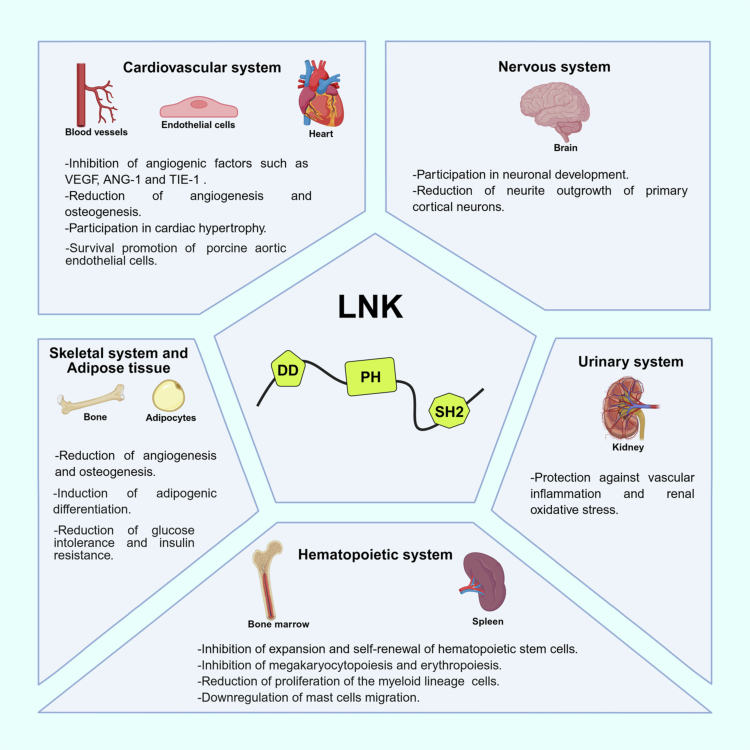
LNK activity in different biological systems. LNK participates in a diverse range of physiological activities in the cardiovascular, nervous, urinary, hematopoietic, skeletal, and adipose systems. Created in https://BioRender.com.

According to RNA expression data from the Human Protein Atlas (https://www.proteinatlas.org),^39^ LNK is mainly expressed in the bone marrow, spleen, and lymph nodes, but is also found in the brain, lung, colon, female reproductive system (vagina, ovary, fallopian tube, endometrium, cervix), and breasts. Most LNK functions in the hematopoietic system have been defined based on phenotypic analysis of *Sh2b3*-deficient mice^11^^,^^40^ and the identification of *SH2B3* mutations in hematological malignancies.^41^ However, it is important to note that LNK inhibitory activity has also been described in non-hematopoietic systems, including the cardiovascular (heart, blood vessels), urinary (kidney), nervous (brain), and skeletal (bone) systems (Figure 2). Therefore, here we will focus on describing what is currently known about LNK function in some of these systems in a physiological context, as this may provide insight into LNK dysfunction in certain solid tumors.

### LNK in the cardiovascular and skeletal systems

In the cardiovascular system, LNK activity has been detected in both cardiac cells and ECs.^42^^,^^43^ Analysis of human and rodent cardiac tissue demonstrated a significant increase in LNK expression during pathological cardiac remodeling. Moreover, LNK has been shown to induce cardiac hypertrophy by activating FAK and AKT, leading to phosphorylation of GSK-3β and mTOR.^42^

In addition, LNK has been reported to play an essential role in vascular remodeling. In a murine model, *Sh2b3* deficiency was associated with upregulation of angiogenic factors such as VEGF, ANG-1, and TIE-1, which resulted in enhanced neovascularization.^44^ At the cellular level, LNK plays a significant role in the integrin pathway, which promotes EC migration.^12^ When the integrin β1 subunit recognizes components of the extracellular matrix, a set of proteins is recruited to focal adhesions, including LNK.^12^ The presence of LNK allows activation of integrin-linked kinase and subsequent phosphorylation of AKT and GSK-3β, which stimulates integrin signaling and improves cell adhesion, thus decreasing migration (Figure 1B).^12^ Indeed, an increase in LNK expression dramatically enhances the number of focal and cell matrix adhesions.^12^

LNK overexpression in ECs decreases TNF-α–induced VCAM-1 expression by inhibiting ERK activation and promoting AKT activation.^45^ Furthermore, in porcine aortic ECs, LNK overexpression reduces the rate of TNF-mediated anoïkis.^43^ In contrast, LNK deficiency promotes EC dysfunction, oxidative stress, and inflammation in the kidney, suggesting a potential anti-inflammatory role for LNK.^46^ Likewise, LNK deficiency fosters greater angiogenesis/vasculogenesis and osteogenesis in mice with bone fractures, suggesting that LNK may facilitate osteoblast terminal differentiation.^47^

### LNK in the nervous system

Little is known about LNK expression in the nervous system. However, it appears to be expressed during rat brain development.^48^ A study performed in an *in vitro* neural differentiation model showed that *SH2B3* overexpression inhibited neural growth factor–induced differentiation and decreased neurite outgrowth of primary cortical neurons by blocking activation of the PLCγ, MEK/ERK, and PI3K/AKT pathways.^48^ More recently, Kaur et al. showed that the *SH2B3* transcript is part of a gene network that regulates neuronal development.^49^ It is likely that the role of LNK in the nervous system is associated with regulation of the neurotrophin signaling cascade^49^; however, further research is still needed to elucidate the precise functions of LNK in the brain.

### LNK in adipose tissue

Additional LNK functions have been described in animal models of adipogenesis. Using mesenchymal stem cells from *Sh2b3*-deficient mice, Lee et al. demonstrated that LNK regulates adipogenic differentiation through insulin-like growth factor-1/AKT/peroxisome proliferator-activated receptor gamma signaling.^13^ Furthermore, another study demonstrated that *Sh2b3*-deficient mice exhibit greater glucose intolerance and insulin resistance.^50^ Interestingly, this effect was associated with the activation and expansion of adipose group 1 innate lymphoid cells, which reduces the risk of diabetes.^50^ In contrast, little is known about LNK activity in human adipose tissue. An observational study of a heterogeneous population including patients with fallopian tube disorders, pelvic disease, gastrointestinal cancer, and extreme obesity showed that LNK expression in adipose tissue was positively correlated with serum glucose and insulin levels in patients with obesity.^51^ However, the study did not investigate mechanisms regulated by LNK in human adipose cells.

## LNK functions in solid tumors

As explained above, LNK inhibits normal human cell proliferation and migration, notably in the context of hematological disorders.^18^^,^^52^ For this reason, LNK has been proposed to function as a tumor suppressor in hematopoietic cells. However, the role of LNK in solid tumors has been more challenging to define, partly due to cell heterogeneity and the high level of dysregulated signaling within solid tumors. These factors, together with various *SH2B3* genetic variants and different levels of LNK expression, may result in LNK playing different roles across cancer types.

### LNK’s inhibitory functions in renal, lung, and colorectal cancer

As previously stated, the level of LNK expression varies among cancer types. *SH2B3* expression was markedly lower in renal cancer–derived cell lines than in normal HK2 cells. Interestingly, one study identified an eight-gene signature, including *SH2B3*, that was linked to prognosis and immune infiltration in renal clear cell carcinoma, and subsequently validated the expression of these genes *in vitro* (Table 1).^53^ *SH2B3* expression was markedly higher in normal HK2 cells compared with renal cancer cell lines, at both the mRNA and the protein level, supporting its biological relevance in this context.^53^ However, its specific role in renal cancer is unknown.

**Table 1.** Expression and effect of LNK in solid tumors.

Solid tumor	LNK Expression	Effect	References
*Breast cancer*	**Patients** Increased protein levels in triple negative breast cancers (TNBC) compared to non-TNBC tumors.	ND	[22]
	**Cell lines** Increased mRNA and protein levels in TNBC cell lines compared to non-TNBC.	▪ Overexpression of LNK increases: -Proliferation and migration. -Phosphorylation of ERK1/2, STAT3, STAT5 and AKT. -Proliferation and lung metastasis in a xenograft mouse model.	
	**Cell lines** ND	▪ Overexpression of KAT2A increases LNK protein levels. ▪ LNK overexpression: -Increases proliferation, migration and invasion. ▪ Silencing of LNK: -Decreases proliferation, migration and invasion. -Reduces half‐life of vimentin and decreases cytoskeletal extensions. -Enhances carboplatin sensitivity	[54]
*Glioblastoma*	**Patients** Increased mRNA in high grade tumors compared to glioma (grades II and III).	High levels of LNK are associated with poorer survival.	[23]
	**Cell lines** Increased mRNA and protein levels in tumor spheres compared to monolayer cells.	▪ Silencing of LNK: -Decreases proliferation and migration. -Increases apoptosis and sensitivity to temozolomide (TMZ). -Inhibits STAT3 activation. ▪ Knockdown of LNK: -Decreases pSTAT3. -Increases TMZ sensitivity in a mouse xenograft model and suppresses tumor growth.	
*Melanoma*	**Patients** Increased mRNA and protein levels in primary tumors compared to normal samples.	High levels of LNK are associated with poorer survival.	[24]
	**Cell lines** High mRNA levels in melanoma cell lines compared to other cancer cell lines.	▪ High levels of LNK levels are correlated with hyperactivation of RAF/MAPK pathway. ▪ LNK overexpression: -Increases resistance to anoïkis due to the decrease in levels of cleavage caspase 3/9, cleavage PARP and BIM. -Attenuates IFN-*γ*-induced expression of MHC class I and class II genes. ▪ Silencing of LNK: -Increases IRF1 and PD-L1 protein levels. -Enhances tumor-suppressive effect of PD-1 antibody in a mouse model.	
*Ovarian cancer*	**Patients** Increased mRNA and protein levels in tumor compared to normal tissue.	High LNK levels decrease overall survival.	[25]
	**Cell lines** Protein levels increased during serum starvation in ovarian cancer cell lines.	▪ Overexpression of LNK: -Increases cell survival in starving conditions and in the presence of cytotoxic drugs. -Increases phosphorylation of AKT, JNK1/2 but decreases pJAK2. -Decreases migration. ▪ Silencing of LNK decreases tumor size and weight in murine xenograft models.	
*Thyroid cancer*	**Patients** Increased mRNA and protein levels in tumor compared to normal tissues.	ND	[26]
	**Cell lines** Increased protein levels in cancer cell lines compared to a normal human thyroid epithelial cell line.	▪ Silencing of LNK decreases proliferation and the effect depends partially on its interaction with 14-3-3. ▪ Overexpression of LNK: -Increases protein levels of 14-3-3, Bcl-2 and Bcl-xL; and phosphorylation of AKT. -Decreases protein levels of caspase 7/9.	
*Colorectal carcer*	**Patients** Low mRNA and protein levels in cancer tissues compared to normal tissues.	Low expression of LNK is associated with local invasion in patients.	[28]
	**Cell lines** ND	Overexpression of LNK, decreases cell invasion.	
*Kidney cancer*	**Cell lines** Low mRNA and protein levels in renal cancer cell lines compared to a normal human renal tubular epithelial cell line.	ND	[53]
*Lung cancer*	**Patients** Low mRNA and protein levels in tumor tissues compared to normal tissues.	ND	[27]
	**Cell lines** Low mRNA and protein levels in lung cancer cells compared to a normal lung cell line.	▪ Silencing of LNK decreases: -Number of anoïkis cells. -Protein levels of E-cadherin but increases the levels of *N*-cadherin, slug, vimentin, MMP-2 and MPP-9. ▪ Overexpression of LNK decreases: -Proliferation, migration and invasion. -Protein levels of *N*-cadherin but increases protein levels of E-cadherin. -Phosphorylation of JAK2, STAT3, SHP2, PI3K, and AKT. ▪ TGF-β1 increases *p*-SHP2, Grb2, *p*-PI3K, *p*-AKT but overexpression of LNK reversed these changes. **▪** **LNK inhibits JAK2/STAT3 and Grb2/PI3K/AKT signaling by binding to JAK2 and SHP2, respectively.** ▪ LNK restrains tumor growth and metastasis in vivo in a mouse xenograft model.	
	**Patients** *SH2B3* expression was decreased in lung cancer compared to normal samples, according to TCGA data	ND	[55]
	**Cell lines** ND	▪ SH2B3 is a direct target for SMYD5 ▪ SMYD5 knockdown upregulates the expression of *SH2B3* and reduces cell migration and invasion.	

ND: Not Determined.

**Figure 3.**
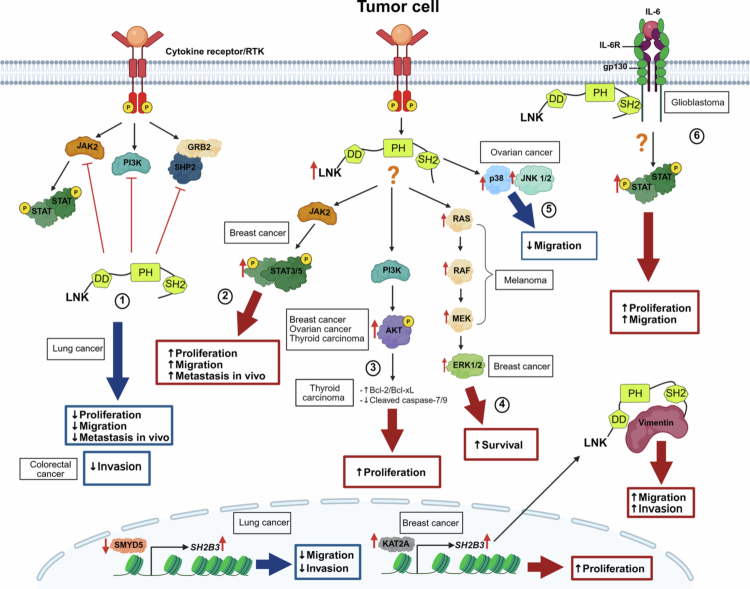
The role of LNK in regulating cell proliferation and migration in solid tumors. (1) In lung cancer, LNK is considered to be a negative regulator of the JAK-STAT, PI3K/AKT, and MAPK pathways. It inhibits JAK2 and interacts with SHP2, reducing cell proliferation, migration, and metastasis in mice. LNK expression appears to be regulated in part by SMYD5 in this type of cancer. In colorectal cancer–derived cell lines, LNK overexpression reduces cell invasion. (2) In breast cancer, high LNK expression levels are associated with increased STAT3 and STAT5 activation, promoting cell proliferation and migration. In addition, LNK expression appears to be influenced by KAT2A, thereby stabilizing vimentin and promoting cell migration. (3) LNK overexpression and AKT hyperactivation have been observed in breast cancer, ovarian cancer, and thyroid cancer. In thyroid cancer, LNK expression increases anti-apoptotic protein expression levels and decreases caspase-7/9 activity. (4) In melanoma, high LNK expression correlates with hyperactivation of the RAF/MAPK pathway. (5) In ovarian cancer, high LNK expression is associated with JNK1/2 and p38 activation and a consequent decrease in cell migration. (6) In glioblastoma, LNK functions as an adapter protein in response to IL-6 signaling. It binds directly to gp130, allowing STAT3 activation and promoting proliferation and migration. Created in https://BioRender.com.

In the case of lung cancer, a pilot study including 40 patients showed that *SH2B3* expression was lower in tumors than in adjacent normal tissue and that low LNK expression levels correlated with poor prognosis (Table 1).^27^ Furthermore, dataset analysis demonstrated that low LNK expression levels were associated with more advanced disease and lymph node metastasis in patients with lung cancer. To further investigate the significance of LNK expression levels, Wang et al. analyzed lung cancer–derived cell lines overexpressing LNK in a mouse xenograft model. The authors found that high LNK expression levels suppressed cell proliferation.^27^ Furthermore, they demonstrated that LNK bound to JAK2 kinase and SHP2 tyrosine phosphatase, thereby inhibiting their activity and activation of GRB2, PI3K, and AKT, suggesting that LNK controls cell proliferation by directly inhibiting the JAK2/STAT3 and SHP2/GRB2/PI3K/AKT signaling cascades (Figure 3). In addition, knocking down LNK expression promoted cell migration and invasion and the expression of epithelial–mesenchymal transition markers (Figure 3).^27^

LNK has been previously reported to regulate SHP2 activity in B-cell lymphoma,^56^ suggesting that the LNK–SHP2 interaction may be common to hematological and some solid cancers. Moreover, since SHP2 has been reported to activate the JAK/STAT^57^ and MAPK^58^ signaling pathways in some types of cancer and inhibit JAK/STAT signaling in others,^59^^,^^60^ we hypothesize that the LNK–SHP2 association could contribute to the contrasting functions reported for LNK in different solid tumors.

These findings highlight SH2B3 as a central regulator of lung cancer progression. Complementary evidence indicates that, at least in lung cancer cells, LNK is subject to epigenetic regulation. Specifically, Tae et al. identified SMYD5, a methyltransferase, as a direct transcriptional regulator of *SH2B3*, and SMYD5 knockdown upregulated *SH2B3* expression, thereby reducing cell migration and invasion in lung cancer cell lines (Table 1) (Figure 3).^55^ Based on this, the authors proposed that targeting SMYD5 could modulate *SH2B3* expression as a potential strategy for limiting metastasis in this type of cancer.^55^

Regarding colorectal cancer, analysis of 32 patient samples showed that LNK expression was significantly lower in tumor tissue compared to that in normal adjacent tissues (Table 1).^28^ Moreover, the same study showed that lack of LNK expression correlated with tumor invasion, while inducing LNK overexpression in colorectal cancer–derived cell lines reduced their capacity for invasion (Figure 3).^28^ However, the study did not evaluate the molecular mechanisms involved in these LNK-mediated effects.

### LNK in breast cancer

Currently, little is known about the potential role of LNK in breast carcinogenesis. Lv et al. performed immunohistochemical evaluation of LNK expression in tumor tissue samples and found higher LNK expression in more advanced triple-negative tumors compared with non–triple-negative cases.^22^ Consistent with this, cell lines derived from triple-negative tumors showed significantly higher levels of LNK expression than those derived from HER2- and estrogen receptor–positive breast tumors (Table 1). Furthermore, inducing LNK overexpression increased cell proliferation, while silencing LNK resulted significantly inhibited cell growth. Interestingly, LNK overexpression activated ERK1/2 kinases in all cell lines analyzed, and this activation was dependent on either STAT3 or STAT5, depending on the cell line (Figure 3).^22^ Furthermore, inducing LNK overexpression in triple-negative breast cancer–derived cell lines increased cell migration and promoted the formation of lung metastases in a murine xenograft model. Again, LNK's effect on cell migration was associated with activation of the ERK1/2 and STAT5 signal transduction pathways (Figure 3).^22^ Genomic studies with large sample sizes are needed to define the role of LNK in breast cancer.

A more recent study demonstrated that LNK binds to and stabilizes vimentin, promoting EMT and metastasis in triple-negative breast cancer (Table 1).^54^ Furthermore, the authors found that *SH2B3* transcription was regulated by lysine acetyltransferase 2 A (KAT2A). Interestingly, inhibiting the *KAT2A/SH2B3* axis increased the carboplatin sensitivity of triple-negative breast cancer cells.^54^ Together, these observations are consistent with the idea that LNK promotes metastasis in triple-negative breast cancer.

### LNK in glioblastoma

Glioblastoma is another type of cancer in which high levels of LNK have been associated with tumor progression.^23^ It is the most aggressive brain tumor in adults and is commonly linked with aberrant STAT3 activity.^23^ Analysis of transcriptomic datasets showed that *SH2B3* was highly expressed in advanced glioblastomas compared to early-stage gliomas. Consistent with this, elevated *SH2B3* expression levels were associated with poor survival.^23^ Moreover, silencing *SH2B3* expression in glioblastoma-derived cell lines significantly inhibited cell proliferation and migration/invasion, both *in vitro* and in a xenograft mouse model (Table 1). Surprisingly, the positive effects resulting from LNK inhibition were associated with STAT3 activation.^23^ This observation contradicts the purported role of LNK as a negative regulator of the JAK/STAT signaling cascade. However, the authors of the study found that, at least in glioblastoma cells, LNK can interact with the IL-6 receptor subunit gp130, which forms a complex with interleukin 6 receptor (IL-6R), resulting in downstream STAT3 activation (Figure 3).^23^ IL-6 is a pro-inflammatory cytokine, and aberrant IL-6/STAT3 signaling has been associated with various types of cancer.^61^ Cai et al. proposed an alternative mechanism to explain the potential oncogenic effect of LNK in glioblastoma.^23^ However, it remains unclear how the LNK adapter mediates STAT3 activation to promote the progression of glioblastoma and other IL-6–driven solid tumors.

### LNK in melanoma

Cutaneous melanoma responds poorly to traditional chemotherapy but well to immune checkpoint blockade–based immunotherapy.^24^ Genomic analysis of *SH2B3* transcript levels, as well as immunohistochemical evaluation of LNK expression, showed significantly elevated LNK expression in samples from patients with melanoma (Table 1). Increased LNK expression was associated with aberrant activation of the RAS–RAF–MEK signaling pathway; however, the LNK binding partner that mediates this activation is still unknown (Figure 3).^24^ In contrast with observations in breast cancer and glioblastoma, LNK only had a modest effect on melanoma cell line proliferation. However, LNK promoted melanoma cell survival by inhibiting pro-apoptotic signaling triggered by interferon (IFN).^24^ *In vitro* analysis demonstrated that treating melanoma cells with INF-*γ* activates STAT1, which induces *SH2B3* transcription, establishing a negative feedback loop that controls the IFN-*γ* signaling cascade.^24^ These observations are relevant in the context of melanoma immunotherapy, because downregulation of IFN-*γ* signaling is a known strategy for escaping immunosurveillance and abolishing the effect of anti-PD-1 antibody–based immunotherapy.^62^ Thus, LNK could be a useful therapeutic target for improving melanoma immunotherapy.

### LNK in ovarian cancer

The case of ovarian cancer deserves particular attention because, in contrast to colorectal and lung cancer, *in silico* analysis showed that higher LNK expression levels were associated with more advanced disease and worse overall survival than lower LNK expression levels (Table 1).^25^ Interestingly, inducing LNK overexpression in ovarian cancer–derived cell lines resulted in significant inhibition of cell migration, accompanied by enhanced cell attachment.^25^ LNK overexpression was associated with activation of the AKT, mTOR, ERK1/2, JNK1/2, and p38 signaling cascades in both cell lines and tumor samples (Figure 3).^25^ However, the experimental data obtained so far do not fully explain why LNK expression is elevated in advanced ovarian cancer cases. Moreover, Ding et al. found that LNK promotes cell proliferation in eight ovarian cancer-derived cell lines and tumor growth in a murine model; this suggests that proliferation may be associated with the phosphorylation of PDK1, AKT, p38, and JNK1/2, which are critical molecular signals for cell proliferation (Figure 3).^25^ Furthermore, the authors demonstrated that 14-3-3 can regulate LNK activity in ovarian cancer.

### LNK in thyroid carcinoma

LNK has also been studied in thyroid carcinoma.^26^ LNK expression was initially evaluated in 11 tissue samples including anaplastic thyroid carcinoma, differentiated thyroid cancer, and normal thyroid tissue (Table 1). Real-time PCR analysis showed that *SH2B3* mRNA expression was higher in anaplastic thyroid carcinoma than in differentiated thyroid cancer or normal thyroid tissue.^26^ These results correlated with LNK levels detected by immunohistochemistry.^26^ Although the study only involved a small number of cases, the difference in LNK expression that was observed suggests a potential association not only with thyroid cancer, but also with clinical aggressiveness, because differentiated thyroid cancer tends to grow more slowly. In contrast, anaplastic thyroid carcinoma tends to grow faster than other types of malignant thyroid neoplasms.^26^ Thus, the authors further studied the role of LNK in thyroid cancer cell proliferation using thyroid cancer–derived cell lines. Inhibiting LNK expression significantly reduced the proliferative capacity of the cell lines.^26^ The authors also demonstrated that inducing LNK overexpression increased cell proliferation, activated the AKT signaling pathway, and promoted the expression of a series of anti-apoptotic proteins (Figure 3); these effects could create an imbalance between cell proliferation and death, which is a hallmark of thyroid cancer.^26^

## Potential factors contributing to the dual role of LNK in solid tumors

Altogether, the published data seem to suggest that LNK activity is tumor type–dependent. Thus, LNK may participate in divergent molecular processes, such as inhibition or activation of signaling pathways associated with cell proliferation, migration, and invasion, in different tissue backgrounds. At the moment, there is no straightforward explanation for the apparently antagonistic functions of LNK. Nevertheless, the experimental evidence suggests that a number of factors might account for the dual effects of LNK in solid tumors.

### Genetic factors

Although multiple *SH2B3* variants have been described in hematopoietic disorders,^41^ only a few studies have explored whether these variants, or novel ones, affect LNK function or subcellular location in solid tumors. Surprisingly, a review of the role of LNK in modulating JAK/STAT signaling in hormone receptor–positive breast cancer reported that only 0.5% of patients had one or more mutations in *SH2B3*.^63^ This suggests that somatic *SH2B3* mutations in this cancer type are uncommon and may not contribute to LNK-specific functions in this subtype of breast carcinoma.

In contrast, a meta-analysis conducted in 2015 showed that the *SH2B3* polymorphism rs3184504 was associated with colorectal cancer and endometrial cancer.^64^ rs3184504 results in substitution of a tryptophan residue (T allele) for an arginine residue (C allele) at amino acid 262, which is located in the PH domain. The study identified the C allele as the risk allele for cancer,^64^ whereas the T allele was linked to autoimmune disease.^65^ This finding is consistent with two independent studies showing that the rs3184504 locus was associated with lung, colorectal, breast,^66^ and endometrial cancer.^67^ In addition, another study suggested that rs3184504 is linked to breast and ovarian cancer risk in *BRCA1/2* mutation carriers.^68^ As the polymorphism lies within the PH domain, which mediates the association of LNK with the inner face of the cell membrane, it may be more than just a risk factor. In fact, it has been suggested that the presence of the rs3184504 polymorphism could modify LNK subcellular localization and function.

### Aberrant spatiotemporal localization of LNK

The intracellular localization of LNK in solid tumors is not well characterized. In glioblastoma cell lines, LNK co-immunoprecipitates with the IL-6/gp130 receptor. This finding was confirmed in patient-derived samples, where LNK co-localized with the IL6/gp130 receptor at the cytoplasmic membrane.^23^ This suggests that LNK localization in glioblastoma favors its interaction with target molecules such as JAK2. In sharp contrast, a different study reported that, in ovarian cancer cell lines, LNK primarily localizes to the cytoplasm, and to a lesser extent to the nucleus.^25^ This observation opens completely new avenues for LNK research. On the one hand, the presence and potential functions of LNK in the nuclear compartment have not been explored; and on the other hand, cytoplasmic expression of the protein might be associated with a potential inhibitory mechanism, based on the fact that the natural LNK repressor, 14-3-3, is a cytoplasmic protein. 14-3-3 recognizes phosphorylated serine residues in LNK, and GSK-3β phosphorylates LNK serine residues.^33^ Thus, constitutive localization in the cytoplasm in solid tumors may favor hijacking of LNK by 14-3-3, thereby inhibiting its function.

### LNK and SHP2

As mentioned previously, LNK binds to JAK2 and also to SHP2 in lung cancer cells,^27^ which suggests that LNK may regulate both the JAK2/STAT3 and SHP2/Grb2/PI3K/AKT pathways.^27^ LNK-mediated regulation of JAK2 is well-documented. Nevertheless, the interaction between LNK and SHP2 has only been described in hematopoietic cells. The importance of this association in solid cancers lies in the fact that SHP2 is itself a dual-function phosphatase, with opposing effects in cell signaling. For instance, SHP2 can stimulate the MAPK pathway by linking RTKs and Grb2, leading to ERK activation.^58^ However, SHP2 can also dephosphorylate Sprouty, which negatively regulates Grb2, inhibiting the signaling cascade.^58^ Therefore, the association between LNK and SHP2 could be an additional factor contributing to the observed duality of this adapter protein’s functions in some tumors.

## LNK as a potential therapeutic target in solid tumors

Currently, there are no commercially available drugs that target LNK. However, JAK2 inhibitors such as ruxolitinib could have an effect on LNK in hematologic malignancies.

Ruxolitinib is an FDA-approved drug for Philadelphia-negative myeloproliferative neoplasms with driver mutations in *JAK2*, *CALR,* or *MPL* that result in constitutive activation of the JAK/STAT pathway.^69^ In a recent study investigating the importance of LNK in juvenile myelomonocytic leukemia (JMML), the authors reported the co-occurrence of somatic or germline *SH2B3* mutations with *PTPN11* mutations, which are considered to be driver mutations, in a small group of patients with JMML.^70^ The study showed that *SH2B3* mutations produced a truncated protein or affected the SH2 domain.^70^ Using an *in vitro* model, the authors demonstrated augmented activation of STAT5 and ERK in double mutant cells.^70^ These cells showed increased sensitivity to ruxolitinib compared to wild-type cells. Importantly, one patient carrying mutations in *SH2B3* and *PTPN11* who was treated with ruxolitinib exhibited a decreased white blood cell count and resolution of splenomegaly after 10 d of treatment, whereas another patient carrying an *SH2B3* mutation who was treated with ruxolitinib exhibited a rapid reduction in splenomegaly before hematopoietic cell transplantation.^70^ Thus, JAK/STAT inhibitors could represent a promising therapeutic option for certain types of cancer with LNK alterations.

The effect of JAK inhibitors, as part of combination therapy for solid tumors is currently under investigation. Although reduced cell growth has been observed in bladder, cervical, colorectal, and breast cancer cell lines treated with ruxolitinib,^71^ this treatment is not as effective in patients. Some studies have reported favorable results with ruxolitinib, whereas others have shown unexpected or only partial outcomes. A review of preclinical studies and clinical trials reported that ruxolitinib combined with afatinib resulted in partial responses in patients with non-small cell lung cancer,^71^ while in patients with triple-negative breast cancer, ruxolitinib inhibited STAT3 activation but had no clinical effect.^71^

Interestingly, in a phase II trial evaluating clinical response to the CDK4/CDK6 inhibitor palbociclib in patients with advanced acral melanoma, a lack of clinical benefit was associated with JAK2 deletions and *SH2B3* amplifications.^72^ Based on this, *SH2B3* could be explored as a potential biomarker for evaluating the effect of treatment with ruxolitinib and palbociclib on solid tumors.

## Future directions and conclusions

As the number of cancer cases is expected to increase in the coming years, the search for new therapeutic targets remains a priority. However, solid tumors are highly heterogeneous, and identifying proteins that could serve as therapeutic targets has been a challenge. This review demonstrates that LNK regulates critical cellular functions in solid tumors, and that LNK activity may be tumor type–specific.

LNK appears to have dual effects in solid tumors: in breast cancer, glioblastoma, and thyroid cancer, it promotes cell proliferation and migration; whereas in lung cancer, it inhibits cell proliferation and migration. These opposing effects have been attributed to potential regulation of signaling pathways related to proliferation and migration. Nevertheless, regulation of signaling cascades might not be the only factor explaining this behavior. The intracellular localization of LNK has not been studied extensively. Since LNK is an adapter protein, its intracellular localization is crucial for determining its interactions and functions. In addition, the genetic context, such as the presence of the rs3184504 polymorphism, might not only serve as a risk factor for cancer development, but also affect LNK's capacity to interact with and function effectively at the cell membrane. The implications of LNK intracellular localization for regulating tumor-associated functions remain to be explored.

Moreover, several open questions remain concerning whether LNK participates in other biological functions that are essential for solid tumor metastasis. Studies have demonstrated that LNK plays a vital role in the formation of lamellipodia and filopodia in normal cells. However, the participation of LNK in focal adhesion formation and stabilization, which represent the structural basis of tumor cell migration and metastasis, remains unexplored.

Significant progress has been made in understanding the effect of LNK expression on some tumor models, as presented here. In the future, research into the precise molecular mechanisms underlying LNK’s effects in different tumor types will clarify whether this protein can be used as a potential biomarker, or even as a therapeutic target.

## Disclosure of potential conflicts of interest

No potential conflict of interest was reported by the author(s).

## Acknowledgments

The authors wish to thank Ma. Cecilia Aguilar Zacarías and Lucía B. Brito Ocampo for technical assistance.

All authors have read and agreed to the published version of the manuscript.

## Data Availability

Data sharing does not apply to this article, as no new data were created or analyzed in this study.
